# The different predictive effects of the intensity and proportion of CD20 expression on the prognosis of B‐lineage acute lymphocyte leukemia

**DOI:** 10.1002/jha2.414

**Published:** 2022-03-25

**Authors:** Yun Tian, Xiaojiao Wang, Hao Ai, Xiaodong Lyu, Qian Wang, Xudong Wei, Yongping Song, Qingsong Yin

**Affiliations:** ^1^ Department of Hematology, Henan Institute of Hematology Affiliated Cancer Hospital of Zhengzhou University Henan Cancer Hospital Zhengzhou Henan China

**Keywords:** B‐lineage acute lymphocyte leukemia, CD20, mean fluorescence intensity, prognosis, proportion

## Abstract

The prognostic effects of the CD20 positivity have been studied extensively in B‐lineage acute lymphocyte leukemia (B‐ALL) patients, but the results remain controversial. The aim of this study is to investigate the different predictive effects of the intensity and proportion of CD20 expression on the prognosis for B‐ALL patients by retrospective analysis. The mean fluorescence intensity (MFI) and percentage of CD20 on B‐ALL cells from 206 patients with B‐ALL were dynamically measured by flow cytometry, and their optimal cut‐off values were determined using the receiver operating characteristic curve. Changes in MFI and percentage of CD20 at various time points and their relationship with prognosis were analyzed. We found that a low baseline CD20 MFI or high CD20 proportion was significantly associated with shorter 5‐year overall survival and progression‐free survival, and the combination of these two factors could more accurately predict worse survival for B‐ALL patients. Furthermore, low CD20 MFI or a high CD20 proportion had different predictive effects for ALL patients with different clinical characteristics and could serve as an independent risk factor for adverse prognosis. There were significant decreases in both the intensity and proportion of CD20 after recurrence in the absence of rituximab treatment, particularly with CD20 intensity. Notably, the decrease of CD20 intensity after recurrence indicated a more shortened survival time. Finally, we conclude that a low intensity or high proportion of CD20 expression may be used as an indicator for inferior prognosis for B‐ALL patients. CD20 intensity is more likely to be a more universal biomarker for worse prognosis.

## 1 INTRODUCTION

Acute lymphoblastic leukemia (ALL) is a heterogeneous disease with a bimodal distribution, that is, children and elderly individuals. Although 80% of ALL occurs in children, the consequences can be more severe in adults [1, 2, 3]. The 5‐year overall survival (OS) is up to 90% for children with intensive chemotherapy [1, 2] and only 30%–40% for adults older than 40 years [3]. Once patients have recurrent or refractory disease, the estimated 5‐year OS is approximately 10%, and there is no standard treatment. Currently, abnormal karyotype, poor early efficacy including complete remission (CR) in 4 weeks or minimal residual disease (MRD) status within 3 months, high leukocyte counts, and age greater than 35 years are usually considered to be high‐risk characteristics in ALL [4, 5]. Allogeneic hematopoietic stem‐cell transplantation (allo‐HSCT) may partially improve the prognosis of these high‐risk patients [5]. Therefore, it is necessary to identify more and accurate prognostic biomarkers to screen high‐risk patients.

CD20 is a non‐glycosylated protein expressed on the surface of normal and malignant B cells, and it is expressed in approximately 40%–50% of precursor lymphocytes [6]. CD20 functions as a calcium channel that influences cell cycle progression and differentiation via downstream signaling pathways, resulting in lower expression of proapoptotic proteins, such as Bax/Bak, and overexpression of anti‐apoptotic proteins, such as Bcl‐2.

At present, most studies define CD20 positivity (CD20+) as ≥20% of cells expressing CD20 according to NCCN guidelines [4]. CD20+ is often associated with poor prognosis, but this idea remains controversial [7, 8, 9, 10, 11]. Recent studies have shown that a CD20 expression rate between 10% and 20% is associated with worse prognosis compared with expression of less than 10% [7, 12]. Fortunately, the addition of CD20 monoclonal antibodies such as rituximab to chemotherapy regimens have prolonged the survival of Philadelphia chromosome (Ph)‐negative B‐lineage ALL (B‐ALL) patients who are CD20+ [13]. Additionally, a previous study has shown that fluorescence intensity may better describe the distribution of antigens on leukemia cells than percent positivity [14]. However, little is known about the fluorescence intensity of CD20 expression for prognosis assessments for ALL patients [14].

Thus, this study aims to investigate the predictive effects of the intensity and proportion of CD20 expression on the prognosis of B‐ALL patients.

## 2 METHODS

### 2.1 Patients and data collection

Newly diagnosed B‐ALL patients from January 2014 to September 2020 in the Affiliated Cancer Hospital of Zhengzhou University were retrospectively analyzed. The diagnostic criteria were based on 2016 WHO criteria. Exclusion criteria included history of another malignancy within the previous 5 years, uncontrolled serious illnesses or comorbidities, and human immunodeficiency virus infection.

The data collected included information such as age, gender, complete blood count, bone marrow (BM) aspiration and/or biopsy collection, karyotyping, immunophenotyping by flow cytometry, and genetic mutations. Analysis of cerebrospinal fluid was performed in conjunction with prophylactic intrathecal injection therapy. BM aspiration was performed after each course of treatment to determine treatment response and disease status. This study was approved by the Ethics Committee of the Affiliated Cancer Hospital of Zhengzhou University.

### 2.2 Regimens and responses

ALL patients who were suitable for intensive chemotherapy underwent the CALLG2008 protocol (a protocol developed by the Chinese Acute Lymphoblastic Leukemia Cooperative Group for ALL) [15] including induction, consolidation, maintenance, and central nervous system prophylaxis. Ph‐positive ALL patients were treated with tyrosine kinase inhibitors (TKIs) in addition to routine chemotherapy. Due to China's medical insurance, all patients were treated without anti‐CD20 drugs regardless of CD20 expression or not. Allo‐HSCT was recommended for all patients with high‐risk factors, such as age ≥35 years old, high white blood cells (WBC) count at diagnosis, no CR in 4 weeks, and adverse cytogenetics. Patients who were not treated with allo‐HSCT continued to receive consolidation and maintenance chemotherapy for 2–3 years. Salvage strategies for relapsed patients were decided by doctors and patients.

CR was defined as BM blasts <5% with no evidence of extramedullary disease and with recovery of peripheral blood counts (absolute neutrophil count >1.0 × 10^9^/L, platelet count >100 × 10^9^/L). Recurrence was defined as the reappearance of blasts in the blood or BM (≥5%) or any extramedullary site after CR. OS was defined as the time from disease diagnosis to death or last follow‐up. Progression‐free survival (PFS) was defined as the time from diagnosis to disease progression or disease‐related death.

### 2.3 Calculation of the optimal cut‐off values for the proportion and MFI of CD20 expression

The intensity and proportion of CD20 expression on leukemia cells in B‐ALL patients were obtained by gating nucleated cells using FACSCalibur (Becton Dickinson, San Jose, CA, USA) and by analyzing using Navios Flow Cytometer software (Beckman Coulter, Brea, CA, USA). And CD20 expression was dynamically monitored at different time points, such as diagnosis, after the first induction therapy, the first recurrence, and multiple relapse. The mean fluorescence intensity (MFI) for CD20 expression was determined by dividing the mean CD20 antibody staining intensity by that of the isotype control. We used OS of B‐ALL patients as the discriminator to determine the optimal cut‐off value of MFI and proportion of CD20 expression according to the receiver operating characteristic (ROC) curve. The point on the curve where the sum of sensitivity and specificity reached the maximum, namely Youden's index, was the optimal cut‐off value for the marker. According to the ROC curve, the area under the curve (AUC), 95% confidence interval (CI), and optimal cut‐off value were determined.

### 2.4 Statistical methods

SPSS 25.0 and GraphPad Prism 9.0 software were used for statistical analysis. Categorical data were analyzed with the chi‐squared test, and continuous data were analyzed with the independent samples *t*‐test. Comparison of data not normally distributed was performed using the Mann–Whitney *U* test for comparison of two groups, while the Kruskal–Wallis *H* test was used to compare multiple groups. Median follow‐up time was estimated on OS using the reverse Kaplan–Meier method. Survival analysis and plotting of survival curves were performed using the Kaplan–Meier method and Cox regression model. The log‐rank test was used to compare rates between groups. *p*‐Value <0.05 was considered statistically significant.

## 3 RESULTS

### 3.1 Patient characteristics

A total of 206 patients with newly diagnosed B‐ALL were eligible to be evaluated. The median age was 15 years (range: 1–71 years), and the male/female ratio was 1.12 (109:97). Ninety patients (43.7%) experienced recurrence or disease progression, and 69 patients (33.5%) died. The median follow‐up time was 35 months (range: 6–89 months). Data at diagnosis served as baseline data. The baseline characteristics of the 206 patients are summarized in Table 1. The patients were categorized into the high and the low groups according to the optimal cut‐off value of the MFI and proportion of CD20, respectively. All clinical factors included in the subgroup analysis were not statistically different (all *p*‐values ≥ 0.05) (Table S1).

**TABLE 1:** The baseline characteristics of 206 patient

Variables	No. of patients (%)
Gender	
Male	109 (52.9)
Female	97 (47.1)
Age	
≤14	100 (48.6)
15—35	61 (29.6)
≥35	45 (21.8)
WBC (×10^9^/L)	
<30	149 (72.3)
≥30	57 (27.7)
Hb (g/L)	
<90	124 (60.2)
≥90	82 (39.8)
PLT (×10^9^/L)	
<100	148 (71.8)
≥100	58 (28.2)
CD20 proportion	
Low	61 (29.6)
High	145 (70.4)
CD20 MFI	
Low	87 (42.2)
High	119 (57.8)
Allo‐HSCT	
Yes	30 (14.6)
No	176 (85.4)
Ph	
Positive	41 (19.9)
Negative	165 (80.1)
Gene mutation^a^	
Unmutation	33 (16.0)
1	44 (21.4)
≥2	34 (16.5)
Unknown	95 (46.1)
CR in 4 weeks	
Yes	185 (89.8)
No	21 (10.2)
MRD in 3 months	
Yes	126 (61.2)
No	35 (17.0)
Unknown	45 (21.8)

Abbreviations: Allo‐HSCT, allogeneic hematopoietic stem‐cell transplantation; CR, complete remission; Hb, hemoglobin; MFI, mean fluorescence intensity; MRD, minimal residual disease; Ph, Philadelphia chromosome; PLT, platelet; WBC, white blood cell.

^a^ Gene mutation represents the number of gene mutation sites.

### 3.2 Optimal cut‐off values of the proportion and MFI of CD20 expression

**FIGURE 1:**
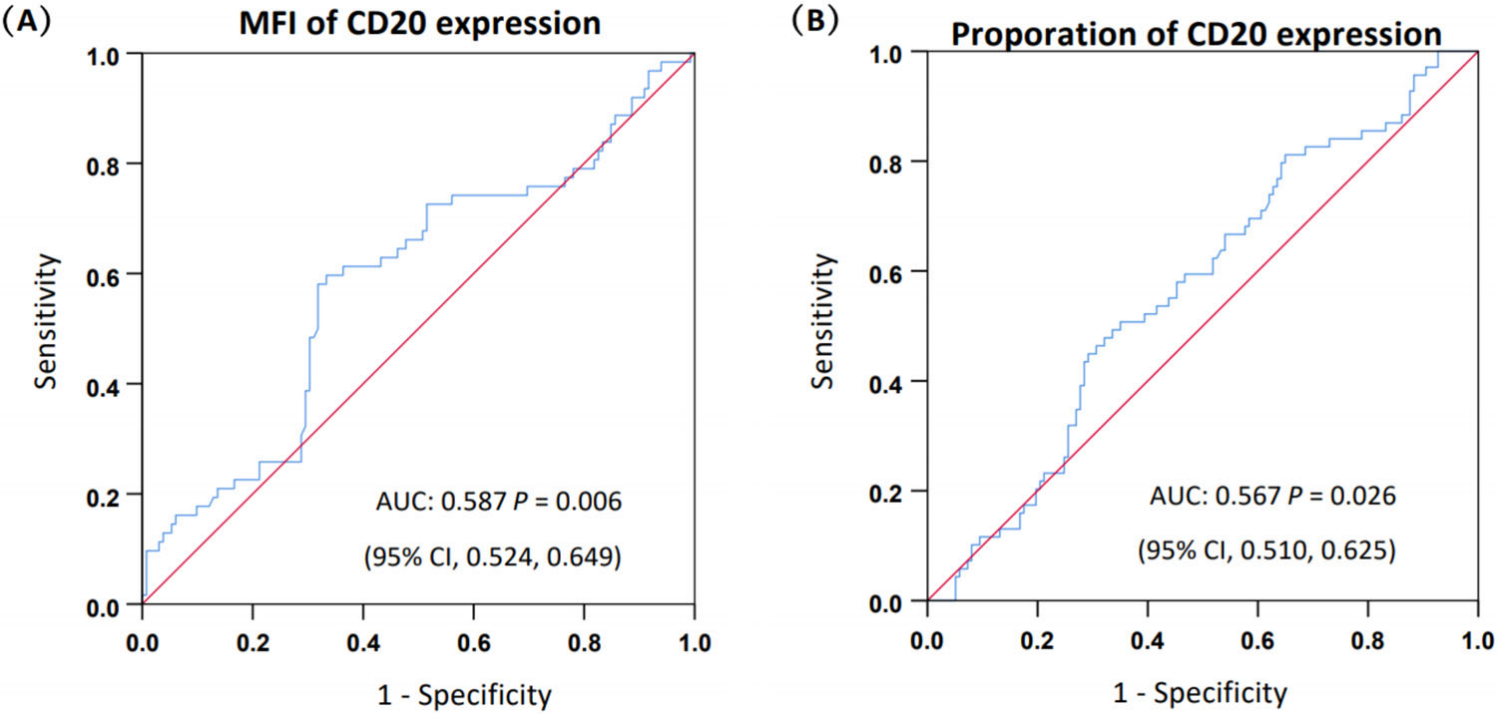
Receiver operating characteristic (ROC) curve analysis of the mean fluorescence intensity (MFI) and proportion of CD20 expression at diagnosis. (A) The optimal cut‐off value of CD20 MFI is 19.98 (area under the curve (AUC) was 0.587; 95% confidence interval (CI): 0.524–0.649, *p* = 0.006). (B) The optimal cut‐off value of CD20 proportion is 11.21% (AUC was 0.567; 95% CI: 0.510–0.625, *p* = 0.026)

The optimal cut‐off value of the MFI for CD20 expression was 19.98, and the AUC was 0.587 (95% CI: 0.524–0.649; *p* = 0.006, Figure 1A). A total of 119 patients (57.8%) had a CD20 MFI ≥19.98, and 87 patients (42.2%) had an MFI <19.98. Similarly, the optimal cut‐off value for the CD20 proportion was 11.21%, and the AUC was 0.567 (95% CI: 0.510–0.625; *p* = 0.026, Figure 1B). A total of 145 patients (70.4%) had a proportion of CD20 expression ≥11.21% at diagnosis, and 61 patients (29.6%) had a CD20 proportion <11.21%.

### 3.3 Baseline low MFI and high proportion of CD20 expression had an adverse effect on the prognosis of B‐ALL patients

**FIGURE 2:**
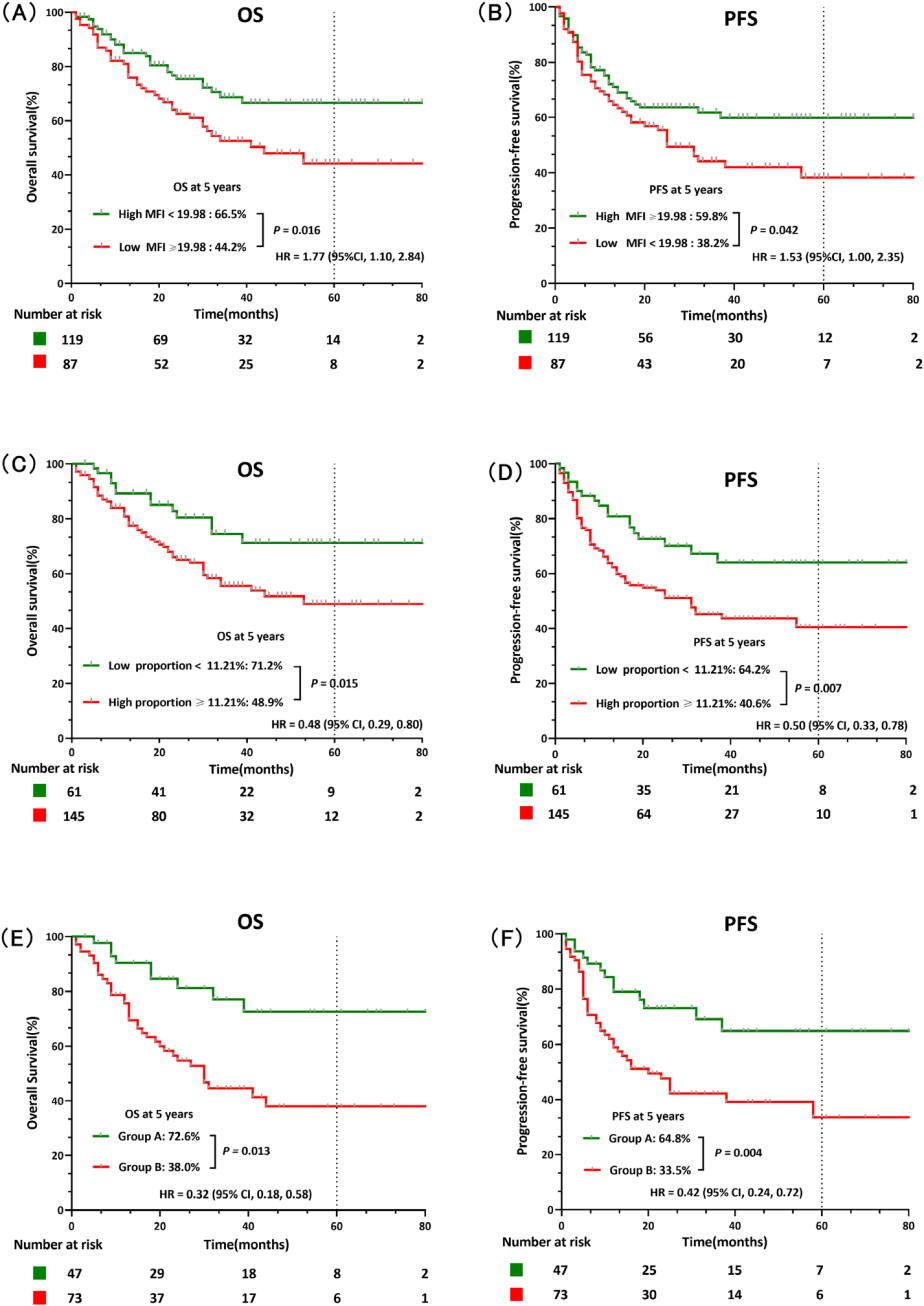
Baseline low mean fluorescence intensity (MFI) and a high proportion of CD20 expression had an adverse effect. (A and B) Low baseline CD20 MFI is remarkably associated with shorter overall survival (OS) and progression‐free survival (PFS) (*p* = 0.016 and 0.042, respectively). (C and D) High baseline CD20 proportion is significantly related to worse OS and PFS (*p* = 0.015 and 0.007, respectively). (E and F) Low MFI combined with high proportion is significantly associated with shorter OS (*p* = 0.013) and PFS (*p* = 0.004)

The 206 eligible patients were categorized into high and low groups according to the optimal cut‐off values for the CD20 MFI or proportion. The 5‐year OS and PFS in the baseline low MFI group were 44.2% and 38.2%, respectively, which were remarkably lower than that in the high MFI group (66.5% and 59.8%; *p* = 0.016 and 0.042, respectively) (Figure 2A,B). Similarly, the 5‐year OS and PFS of patients with a high baseline CD20 proportion were 48.9% and 40.6%, respectively, which were significantly lower than that for patients with a low proportion (71.2% and 64.2%, respectively; *p* = 0.015 and 0.007, respectively) (Figure 2C,D).

In order to further study the superior predictive value of the combination of MFI and proportion on the prognosis of patients with B‐ALL compared with a single index, we screened the patients into two groups: high MFI combined with low proportion group (Group A) and low MFI combined with high proportion group (Group B). The 5‐year OS was significantly lower in Group B (38.0%) compared with Group A (72.6%, *p* = 0.013) (Figure 2E). Similarly, the 5‐year PFS was lower in Group B (33.5%) compared with Group A (64.8%, *p* = 0.004) (Figure 2F). These findings suggest that the combination of the CD20 MFI and CD20 proportion may be used to accurately evaluate the prognosis of B‐ALL patients (Figure S1).

In addition, we investigated the effects of the baseline MFI and proportion of CD20 on early efficacy for B‐ALL patients. It was found that there were no significant differences in the CR rate in 4 weeks or the MRD negative rate within 3 months between the low and high CD20 MFI groups or the low and high CD20 proportion groups (all *p*‐values > 0.05). However, CR rates within 4 weeks were significantly higher in Group A than that in Group B (*p* = 0.042). There was no statistically significant difference in the MRD negative rate within 3 months between the two groups (*p* > 0.05) (Figure S2).

### 3.4 Predictive effects of low MFI and high proportion of CD20 expression on the OS of B‐ALL patients with various clinical characteristics

**FIGURE 3:**
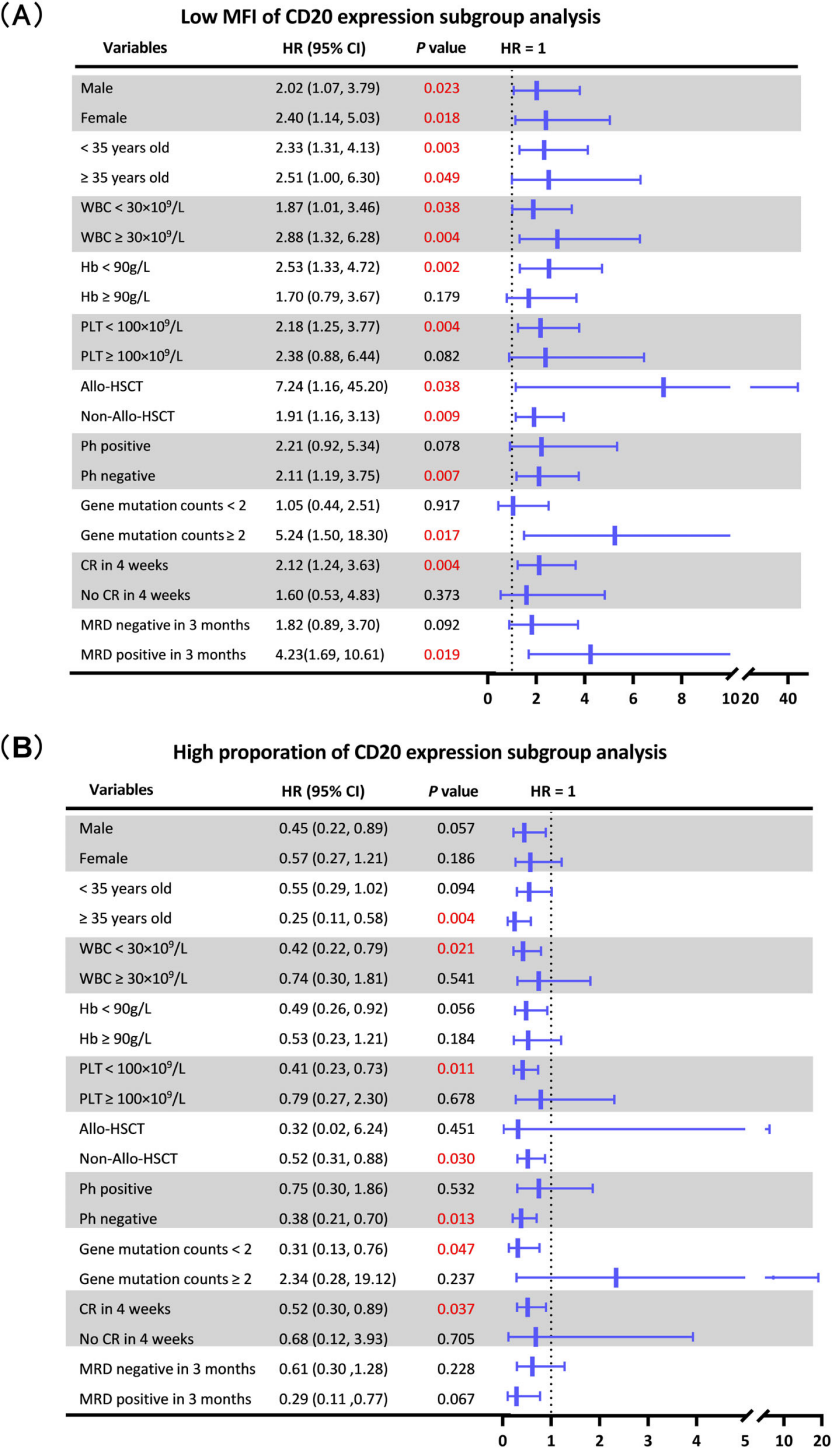
The predictive effects of the mean fluorescence intensity (MFI) and proportion of CD20 expression on overall survival (OS). (A) Lower CD20 MFI is significantly associated with inferior OS for B‐lineage acute lymphocyte leukemia (B‐ALL) patients with a hemoglobin (Hb) <90 g/L, platelets (PLT) <100 × 10^9^/L, Philadelphia chromosome (Ph)‐negativity, ≥2 gene mutations, complete remission (CR) in 4 weeks, and minimal residual disease (MRD) positivity in 3 months, and regardless gender, age, white blood cells (WBC) count, and allogeneic hematopoietic stem‐cell transplantation (allo‐HSCT) (all *p‐*values < 0.05). (B) Higher proportion of CD20 expression is significantly correlated with worse OS for B‐ALL patients who were greater than 35 years, WBC <30 × 10^9^/L, PLT <100 × 10^9^/L, non‐allo‐HSCT, Ph‐negativity, <2 gene mutations, and CR in 4 weeks (all *p‐*values < 0.05)

The CD20 MFI and proportion demonstrated different predictive effects on OS for patients with different clinical characteristics. As shown in Figure 3A, irrespective of age, gender, baseline WBC count, or allo‐HSCT, a lower CD20 MFI suggested shorter survival time for ALL patients (all *p*‐values < 0.05). In addition, lower CD20 MFI was significantly associated with worse OS for B‐ALL patients who had Ph‐negativity, ≥2 gene mutations, CR in 4 weeks, baseline hemoglobin (Hb) <90 g/L, baseline PLT <100 × 10^9^/L, and MRD positivity within 3 months (all *p*‐values < 0.05). However, lower CD20 MFI was not associated with OS for patients with Ph‐positivity, baseline Hb ≥90 g/L, PLT ≥100 × 10^9^/L, MRD negativity within 3 months, or no CR in 4 weeks (all *p*‐values > 0.05).

Similarly, as shown in Figure 3B, a higher proportion of CD20 expression was significantly correlated with worse OS for B‐ALL patients with these factors including greater than 35 years old, Ph‐negativity, and no allo‐HSCT treatment, which were defined as inferior prognosis (all *p*‐values < 0.05). Furthermore, when patients had <2 gene mutations, baseline WBC <30 × 10^9^/L, PLT <100 × 10^9^/L, or CR in 4 weeks, the higher CD20 proportion might predict shorter OS (all *p*‐values < 0.05). However, the high CD20 proportion was not correlated with OS for patients with the characteristics of <35 years old, baseline WBC ≥30 × 10^9^/L or baseline PLT ≥100 × 10^9^/L, allo‐HSCT, Ph‐positive, ≥2 gene mutations, or no CR in 4 weeks, and in patients regardless of gender, Hb level, or MRD status within 3 months (all *p*‐values > 0.05).

### 3.5 The MFI and proportion of CD20 expression significantly decreased after recurrence and had adverse effects on prognosis

**FIGURE 4:**
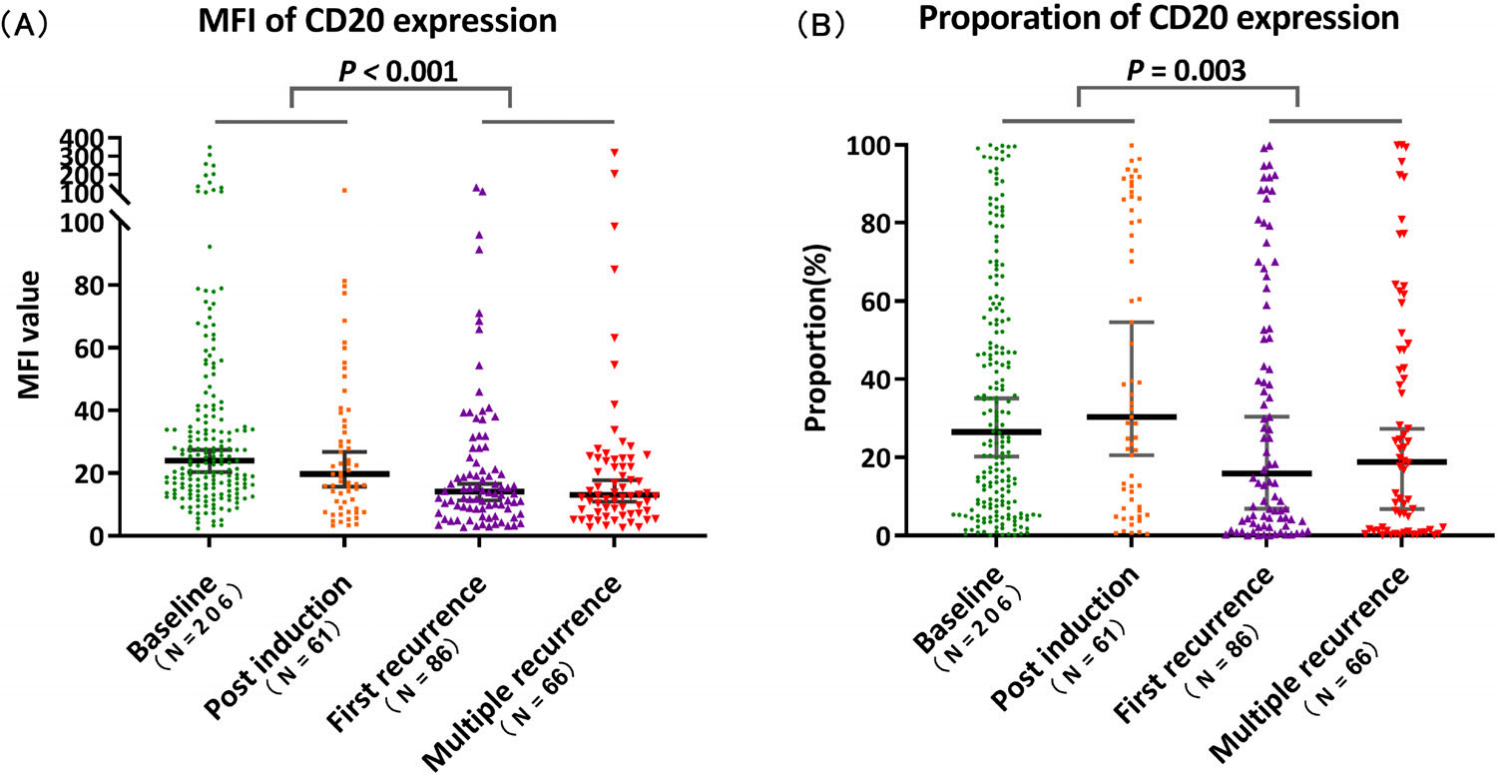
Changes in the mean fluorescence intensity (MFI) and proportion of CD20 in different disease states. There are significant differences in the MFI (*p* < 0.001) and proportion (*p *= 0.019) of CD20 in different disease states. Compared with before recurrence, the MFI (A) (*p* < 0.001) and proportion (B) (*p *= 0.003) of CD20 expression significantly decreased after recurrence. The lines represent the median and 95% confidence interval (CI)

To dynamically measure the levels of MFI and proportion of CD20 at diagnosis, after induction, and after recurrence, we found that there was a significant difference in different disease states (*p* < 0.001 and 0.019, respectively) (Figure 4A,B). The median CD20 MFI was 23.86 at diagnosis, then decreased to 19.66 after induction, further reduced to 14.06 at the first recurrence, and was 13.05 at multiple recurrences. Contrary to the MFI, the baseline median CD20 proportion was 27.12%, and it mildly increased to 30.30% after induction when the MRD was still available; however, once the disease relapsed, it dropped to 15.68% at the first relapse and 18.83% at multiple relapses. Moreover, within‐group comparisons revealed there was a significant decrease in the CD20 MFI (*p* < 0.001) and proportion (*p* = 0.003) after recurrence compared with that before relapse (Figure 4).

**FIGURE 5:**
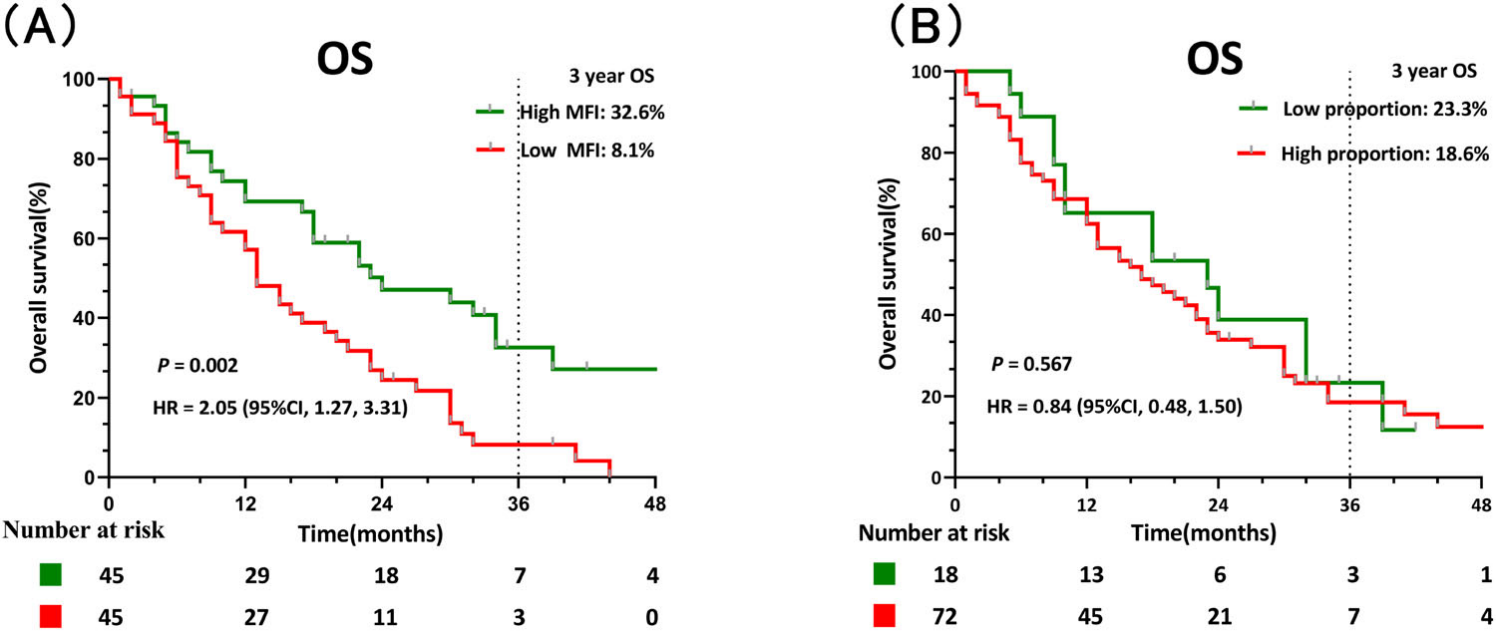
Correlation between CD20 expression after recurrence and overall survival (OS). (A) OS was significantly lower in the low CD20 mean fluorescence intensity (MFI) group than that in the high MFI group after recurrence (*p *= 0.002). (B) The OS in the high CD20 proportion group after recurrence mildly decreased compared with the low proportion group (*p *= 0.567)

To further explore the impact of decreased CD20 expression after relapse on survival time, 90 patients with recurrent ALL were analyzed and grouped according to the optimal threshold of the MFI and proportion of CD20 expression. The 3‐year OS in the low MFI group (8.1%) after recurrence was significantly lower than the high group (32.6%) (*p* = 0.002) (Figure 5A). In addition, the 3‐year OS of patients with a high CD20 proportion (18.6%) after relapse mildly decreased compared with the 23.3% observed for patients with a low CD20 proportion (*p* = 0.567) (Figure 5A).

### 3.6 Low MFI and high proportion of CD20 expression are independent prognostic factors for poor survival

In univariate analysis, low CD20 MFI, high CD20 proportion, ≥35 years old, baseline WBC ≥30 × 10^9^/L, Ph‐positive, MRD positive within 3 months, and no CR within 4 weeks were significant risk factors for poor OS and PFS. Moreover, allo‐HSCT could significantly prolonged the OS (Table 2).

**TABLE 2:** Univariate analysis of risk factors associated with overall survival (OS) and progression‐free survival (PFS)

Variables	OS HR (95% CI)	*p*‐Value	PFS HR (95% CI)	*p*‐Value
Age (<35 versus ≥35)	0.45 (0.24–0.85)	0.002	0.57 (0.29–1.00)	0.018
Gender (male versus female)	1.32 (0.82–2.11)	0.253	1.22 (0.80–1.84)	0.349
WBC (<30 × 10^9^/L versus ≥30 × 10^9^/L)	0.43 (0.24–0.76)	0.000	0.47 (0.28–0.77)	0.000
Hb (<90 g/L versus ≥90 g/L)	0.93 (0.57–1.51)	0.753	0.95 (0.62–1.45)	0.762
PLT (<100 × 10^9^/L versus ≥100 × 10^9^/L)	1.14 (0.67–1.96)	0.635	1.17 (0.74–1.86)	0.507
CD20 MFI (low versus high)	1.77 (1.10–2.84)	0.016	1.43 (1.00–2.35)	0.042
CD20 proportion (low versus high)	0.48 (0.29–0.80)	0.015	0.50 (0.33–0.78)	0.007
Allo‐HSCT (yes versus no)	0.37 (0.20–0.69)	0.025	0.53 (0.30–0.90)	0.058
Gene mutation (<2 versus ≥2)	0.84 (0.39–1.82)	0.638	1.07 (0.56–2.06)	0.836
Ph (negative versus positive)	0.55 (0.30–1.00)	0.019	0.59 (0.34–1.01)	0.036
CR in 4 weeks (yes versus no)	0.44 (0.19–0.99)	0.003	0.35 (0.16–0.79)	0.000
MRD in 3 months (negative versus positive)	0.42 (0.21–0.83)	0.001	0.35 (0.18–0.68)	0.000

Abbreviations: Allo‐HSCT, allogeneic hematopoietic stem‐cell transplantation; CI, confidence interval; CR, complete remission; Hb, hemoglobin; MFI, mean fluorescence intensity; MRD, minimal residual disease; Ph, Philadelphia chromosome; PLT, platelet; WBC, white blood cell.

Statistically significant clinical factors for OS and PFS (*p* < 0.1) were included in the multivariate analysis, revealing that WBC count ≥30 × 10^9^/L, ≥35 years old, low CD20 MFI, and MRD positive within 3 months were independent risk factors for inferior OS. In contrast, allo‐HSCT was a protective factor for long‐term survival. Similarly, baseline WBC ≥30 × 10^9^/L, ≥35 years old, high CD20 proportion, no CR in 4 weeks, and MRD positive within 3 months were independent risk factors for worse PFS (Table 3).

**TABLE 3:** Multivariate analysis of risk factors associated with overall survival (OS) and progression‐free survival (PFS)

Variables	OS HR (95% CI)	*p*‐Value	PFS HR (95% CI)	*p*‐Value
Age (<35 versus ≥35)	0.53 (0.29–0.97)	0.040	0.54 (0.31–0.96)	0.036
WBC (<30 × 10^9^/L versus ≥30 × 10^9^/L)	0.35 (0.20–0.62)	0.000	0.45 (0.27–0.76)	0.003
CD20 MFI (low versus high)	1.95 (1.09–3.49)	0.025	1.14 (0.66–1.99)	0.631
CD20 proportion (low versus high)	0.67 (0.31–1.43)	0.300	0.51 (0.26–0.98)	0.043
Allo‐HSCT (yes versus no)	0.32 (0.12–0.84)	0.020	0.57 (0.27–1.02)	0.132
Ph (negative versus positive)	1.30 (0.68–2.47)	0.428	1.53 (0.86–2.73)	0.152
CR in 4 weeks (yes versus no)	0.59 (0.26–1.34)	0.205	0.38 (0.18–0.77)	0.008
MRD within 3 months (negative versus positive)	0.37 (0.21–0.67)	0.001	0.45 (0.26–0.79)	0.005

Abbreviations: Allo‐HSCT, allogeneic hematopoietic stem‐cell transplantation; CI, confidence interval; CR, complete remission; MFI, mean fluorescence intensity; MRD, minimal residual disease; Ph, Philadelphia chromosome; WBC, white blood cell.

## 4 DISCUSSION

The prognostic effects of the CD20 expression percentage have been studied extensively in B‐ALL patients, but the results remain controversial. In this retrospective study, we focused not only on the CD20 proportion but also on the CD20 intensity and found that their different prognostic values, that is, low CD20 MFI or high CD20 proportion, can be used as an independent indicator of inferior prognosis for B‐ALL patients, and these were applied to ALL patients with different clinical factors. Moreover, the CD20 MFI combined with the CD20 proportion could more accurately evaluate poor prognosis for B‐ALL patients. There were significant decreases in both the intensity and proportion of CD20 expression after recurrence in the absence of rituximab treatment, particularly for the intensity. Notably, the decrease of CD20 intensity after recurrence indicated a more shortened survival time.

In this study, we first determined the optimal thresholds of the proportion and MFI of CD20. Coincidently, the optimal value for the CD20 proportion was 11.21%, which is similar to the optimal value of 11.7% found in a previous study in which the high percentage was an independent adverse factor for event‐free survival [12]. The effects of a high CD20 percentage on the survival of B‐ALL patients has been widely demonstrated [7, 12], and the prognostic effects of CD20 intensity have been less studied [14]. To screen for more prognostic biomarkers for B‐ALL patients, we first determined that >35 years old, high WBC, no allo‐HSCT treatment, no CR in 4 weeks, and MRD positive within 3 months are predictors of inferior outcome, which is consistent with the accepted high‐risk index [3, 4]. More notably, low CD20 MFI and high CD20 proportion were listed as independent poor prognostic factors for OS and PFS. In addition, lower MFI or higher proportion of CD20 was closely associated with shorter survival for newly diagnosed or refractory/relapsed patients. By contrast, patients with high‐intensity CD20 expression hada favorable prognosis.

To specifically elucidate the prognostic value of CD20 expression, we found that the predictive effects of CD20 intensity and CD20 proportion for survival could be applied to ALL patients with different clinical factors. Notably, the prognostic effects of low CD20 MFI can cover a wider range of B‐ALL patients than high CD20 proportion. Interestingly, high proportion or low intensity of CD20 were not associated with OS for Ph‐positive ALL patients. The underlying reason may be due to the integration of TKIs into chemotherapy regimens [3]. Moreover, the low intensity and high percentage of CD20 have similar predictive effects on worse OS for patients who achieve CR within 4 weeks.

Fortunately, as reported in the literature [16, 17], the addition of rituximab to standard chemotherapy can significantly improve the efficacy and prognosis of patients with CD20‐positive lymphoid malignancies. Moreover, rituximab‐mediated effects depend on CD20 levels with higher intensity expression, leading to higher elimination [17, 18]. In addition, some studies have reported that patients with a CD20 expression level below 20% may also benefit from rituximab [7, 12]. In this study, we found that patients with low intensity and high percentage of CD20 had the worst prognosis and may be suitable candidates for the use of higher affinity anti‐CD20 antibodies, such as obinutuzumab or ofatumumab [6].

Generally, CD20 expression was down‐regulated in response to anti‐CD20 therapy because of antigenic modulation and gene mutations [19, 20] or "shaving" caused by the endocytosis of monocytes and macrophages [21, 22]. However, this study found there is a significant decrease both in the intensity and proportion of CD20 expression after recurrence in the absence of rituximab treatment, and the underlying reasons remain unclear. Recent studies have reported that patients with NOTCH1 mutations are chemorefractory and associated with lower CD20 MFI in B‐cell tumors [23, 24, 25]. Moreover, expression of the MS4A1 gene encoding CD20 was negatively regulated by the transcription factors FOXO1, CREM, EZH2, and MYC, and the decrease in CD20 expression was commonly found in relapsed/refractory non‐Hodgkin lymphomas [6, 26, 27, 28, 29]. Furthermore, the decrease in CD20 expression after recurrence may be due to the selective pressure caused by the expansion of chemoresistant subclones [30]. Nevertheless, it appears that decreased CD20 expression may be associated with the worst prognosis and not just CD20 antibody privilege, which may be related to chemoresistance [31].

This study is an extension and supplement for traditional biomarkers. Some of the limitations of this study include its retrospective design and the absence of control studies including treatment with rituximab. Further prospective studies are needed in the future.

Our findings suggest that not only a high CD20 proportion but also a low CD20 intensity are significantly associated with worse OS and PFS for B‐ALL patients. The decrease of CD20 intensity after recurrence indicated a more shortened survival time. It should be noted that CD20 intensity is more likely to be a more universal biomarker for adverse prognosis than CD20 proportion.

## FUNDING INFORMATION

Foundation for Young Teachers’ Basal Research of Zhengzhou University (jc202050015).

## CONFLICT OF INTEREST

The authors declare they have no conflicts of interest.

## ETHICS STATEMENT

The ethics committees of Affiliated Cancer Hospital and Affiliated People's Hospital of Zhengzhou University have approved this study.

## PATIENT CONSENT STATEMENT

Informed consent was obtained from all participants included in the study.

## AUTHOR CONTRIBUTIONS

Yun Tian collected and analyzed the data, and wrote the paper. Xiaojiao Wang, Hao Ai, Xiaodong Lyu, and Qian Wang collected samples and provided clinical data. Xudong Wei and Yongping Song reviewed the final version of the manuscript and assisted in the critical review of the manuscript and data. Qingsong Yin designed the research study, wrote the paper, and provided critical revisions. All authors reviewed the manuscript.

## Supporting information

Supporting Information
