# Supplementary material for: The different predictive effects of the intensity and proportion of CD20 expression on the prognosis of B‐lineage acute lymphocyte leukemia
Source: EJHaem. 2022 Mar 25;3(2):443–52. doi: 10.1002/jha2.414 (PMC9176059; doi:10.1002/jha2.414)
Supplement: Supplementary file 1 — Supporting Information [file JHA2-3-443-s001.docx]

**Supplemental Figure：**


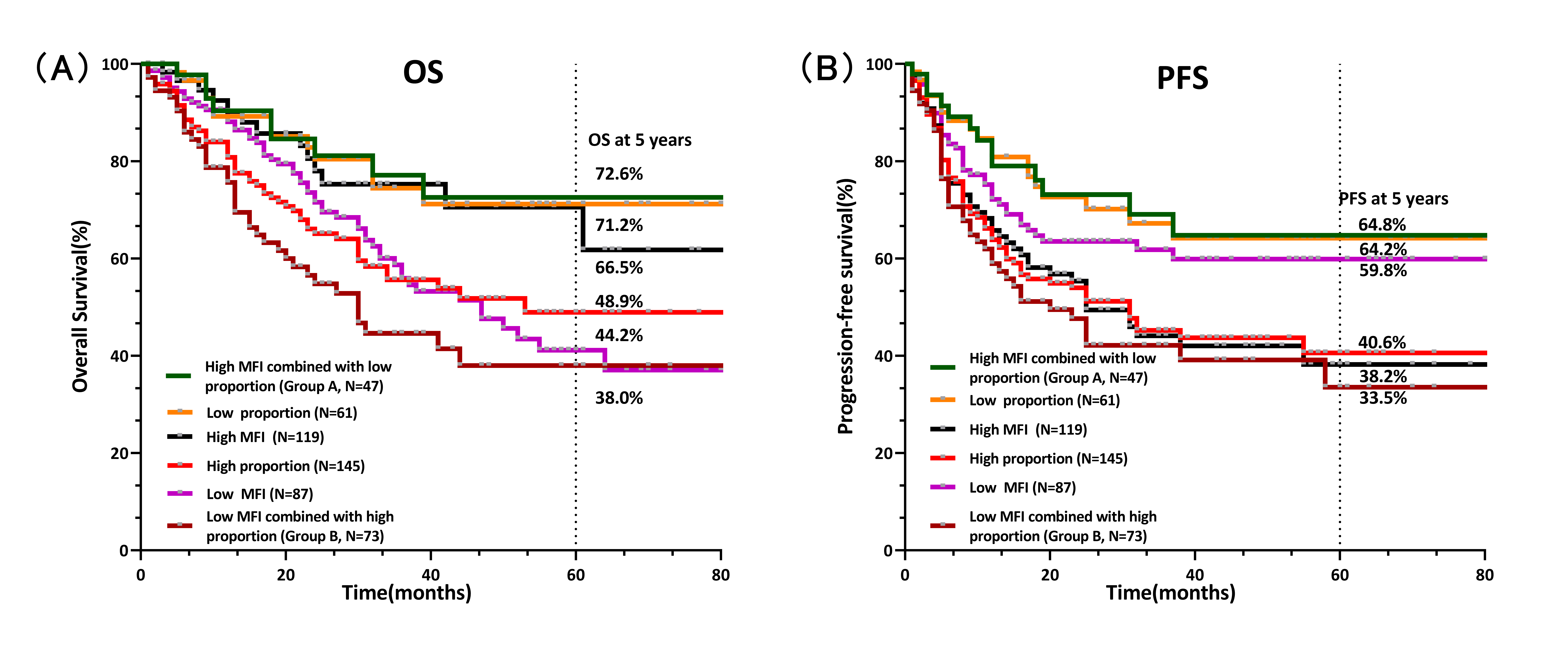


**Supplemental Figure 1** mean fluorescence intensity (MFI) combined with proportion of CD20 have more accurate predictive value than a single index.

(A and B) show the overall survival (OS) and progression‐free survival (PFS) in various groups. High MFI combined with low proportion (Group A) had the best prognosis of all subgroups, while low MFI combined with high proportion (Group B) had the worst prognosis.

**
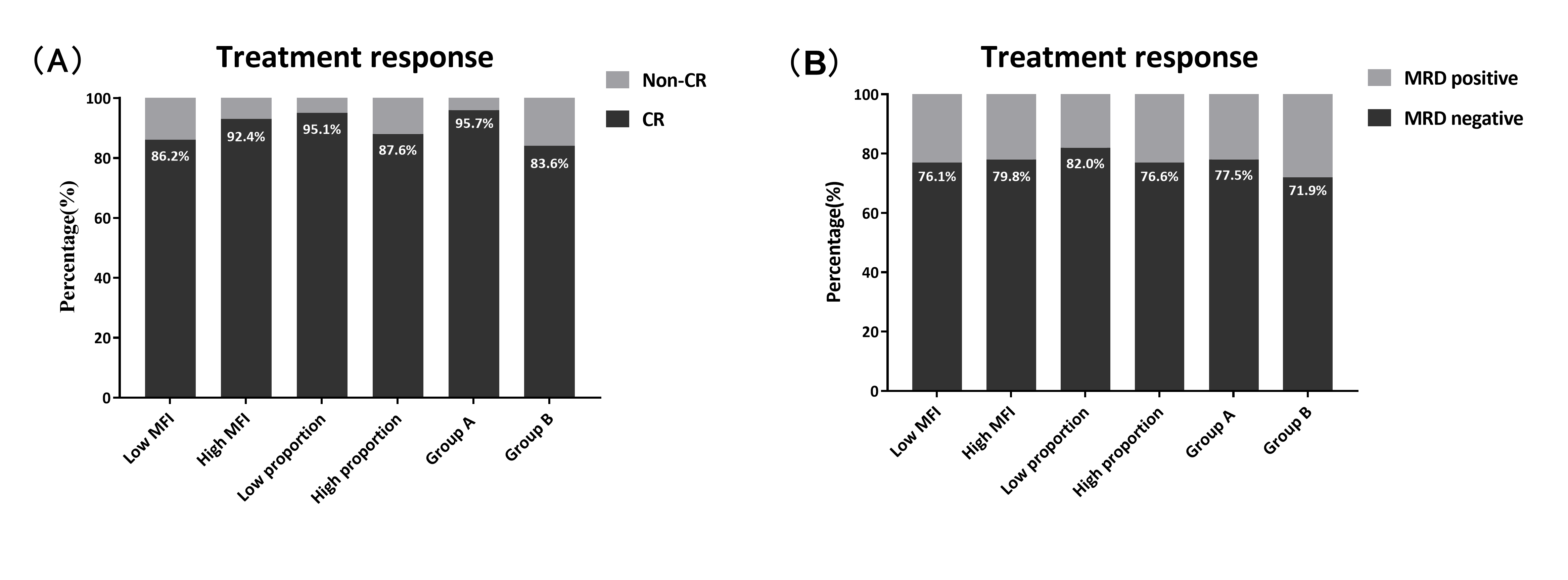
**

**Supplemental Figure 2** The effects of the mean fluorescence intensity (MFI) and proportion of CD20 at diagnosis on early efficacy.

(A) shows the complete remission (CR) in various groups. The CR rate within 4 weeks was 95.7% and 83.6% in groups A and B, respectively. CR rate in group A was significantly higher than that in group B (p = 0.042). (B) shows the minimal residual disease (MRD) negative rate within 3 months in various groups, and there was no statistically significant difference between the groups (All p- values > 0.05).

**Supplementary Table：**

**Supplemental Table：Clinical characteristics of 206 patients with B-ALL**

| **Variables** | **No. (%)** | **CD20 MFI** | | | **CD20 proportion** | | |
| --- | --- | --- | --- | --- | --- | --- | --- |
|  |  | **low** | **high** | ***P* value** | **low** | **High** | ***P* value** |
| **Gender** |  |  |  | 0.391 |  |  | 0.316 |
| male | 109 (52.9) | 43 (20.9) | 66 (32.0) |  | 29 (14.1) | 80 (38.8) |  |
| female | 97 (47.1) | 44 (21.4) | 53 (25.7) |  | 32 (15.5) | 65 (31.6) |  |
| **Age** |  |  |  | 0.088 |  |  | 0.324 |
| <35 | 161 (78.2) | 73 (35.5) | 88 (42.7) |  | 45 (21.9) | 116 (56.3) |  |
| ≥ 35 | 45 (21.8) | 14 (6.7) | 31 (15.1) |  | 16 (7.8) | 29 (14.0) |  |
| **WBC (×10^9^/L)** |  |  |  | 0.543 |  |  | 0.187 |
| <30 | 149 (72.3) | 61 (29.6) | 88 (42.7) |  | 48 (23.3) | 101 (49.0) |  |
| ≥30 | 57 (27.7) | 26 (12.6) | 31 (15.1) |  | 13 (6.3) | 44 (21.4) |  |
| **Hb (g/L)** |  |  |  | 0.333 |  |  | 0.930 |
| <90 | 124 (60.2) | 49 (23.8) | 75 (36.4) |  | 37 (18.0) | 87 (42.2) |  |
| ≥90 | 82 (39.8) | 38 (18.4) | 44 (21.4) |  | 24 (11.7) | 58 (28.1) |  |
| **PLT (×10^9^/L)** |  |  |  | 0.874 |  |  | 0.690 |
| <100 | 148 (71.8) | 62 (30.1) | 86 (41.7) |  | 45 (21.8) | 103 (50.0) |  |
| ≥100 | 58 (28.2) | 25 (12.2) | 33 (16.0) |  | 16 (7.8) | 42 (20.4) |  |
| **Allo-HSCT** |  |  |  | 0.789 |  |  | 0.702 |
| yes | 30 (14.6) | 12 (5.8) | 18 (8.8) |  | 8 (3.9) | 22 (10.7) |  |
| no | 176 (85.4) | 75 (36.4) | 101(49.0) |  | 53 (25.7) | 123 (59.7) |  |
| **Ph** |  |  |  | 0.911 |  |  | 0.274 |
| positive | 41 (19.9) | 17 (8.3) | 24 (11.6) |  | 15 (7.3) | 26 (12.6) |  |
| negative | 165 (80.1) | 70 (34.0) | 95 (46.1) |  | 46 (22.3) | 119 (57.8) |  |
| **Gene mutation**# |  |  |  | 0.367 |  |  | 0.415 |
| <2 | 77 (37.4) | 27 (13.1) | 50 (24.3) |  | 24 (11.7) | 53 (25.7) |  |
| ≥2 | 34 (16.5) | 15 (7.3) | 19 (9.2) |  | 8 (3.9) | 26 (9.7) |  |
| unknown | 95 (46.1) | 45 (21.8) | 50 (24.3) |  | 29 (14.1) | 66 (32.0) |  |
| **CR in 4 weeks** |  |  |  | 0.144 |  |  | 0.105 |
| yes | 185 (89.8) | 75 (36.4) | 110 (53.4) |  | 58 (28.2) | 127 (61.6) |  |
| no | 21 (10.2) | 12 (5.8) | 9 (4.4) |  | 3 (1.5) | 18 (8.7) |  |
| **MRD in 3 months** |  |  |  | 0.671 |  |  | 0.440 |
| yes | 126 (61.2) | 49 (23.8) | 77 (37.4) |  | 41 (19.9) | 85 (41.3) |  |
| no | 35 (17.0) | 15 (7.3) | 20 (9.7) |  | 9 (4.4) | 26 (12.6) |  |
| unknown | 45 (21.8) | 23 (11.1) | 22 (10.7) |  | 11 (5.3) | 34 (16.5) |  |

#Gene mutation represents the number of gene mutation sites. Abbreviations: WBC, white blood cell; Hb, hemoglobin; PLT, Platelet; BM, bone marrow; Allo-HSCT, allogeneic hematopoietic stem-cell transplantation; Ph, Philadelphia chromosome; CR, complete remission; MRD, minimal residual disease.
